# Erythromycin Restores Osteoblast Differentiation and Osteogenesis Suppressed by *Porphyromonas gingivalis* Lipopolysaccharide

**DOI:** 10.3390/ph16020303

**Published:** 2023-02-15

**Authors:** Hikaru Tamura, Tomoki Maekawa, Hisanori Domon, Kridtapat Sirisereephap, Toshihito Isono, Satoru Hirayama, Takumi Hiyoshi, Karin Sasagawa, Fumio Takizawa, Takeyasu Maeda, Yutaka Terao, Koichi Tabeta

**Affiliations:** 1 Division of Microbiology and Infectious Diseases, Graduate School of Medical and Dental Sciences, Niigata University, Niigata 951-8514, Japan; 2 Division of Periodontology, Graduate School of Medical and Dental Sciences, Niigata University, Niigata 951-8514, Japan; 3 Center for Advanced Oral Science, Graduate School of Medical and Dental Sciences, Niigata University, Niigata 951-8514, Japan; 4 Faculty of Dentistry, Chulalongkorn University, Bangkok 10330, Thailand

**Keywords:** macrolides, periodontitis, *Porphyromonas gingivalis* lipopolysaccharide, osteoblastogenesis, developmental endothelial locus-1

## Abstract

The macrolide erythromycin (ERM) inhibits excessive neutrophil accumulation and bone resorption in inflammatory tissues. We previously reported that the expression of developmental endothelial locus-1 (DEL-1), an endogenous anti-inflammatory factor induced by ERM, is involved in ERM action. Furthermore, DEL-1 is involved in the induction of bone regeneration. Therefore, in this study, we investigated whether ERM exerts an osteoblastogenic effect by upregulating DEL-1 under inflammatory conditions. We performed in vitro cell-based mechanistic analyses and used a model of *Porphyromonas gingivalis* lipopolysaccharide (LPS)-induced periodontitis to evaluate how ERM restores osteoblast activity. In vitro, *P. gingivalis* LPS stimulation suppressed osteoblast differentiation and bone formation. However, ERM treatment combined with *P. gingivalis* LPS stimulation upregulated osteoblast differentiation-related factors and *Del1*, indicating that osteoblast differentiation was restored. Alveolar bone resorption and gene expression were evaluated in a periodontitis model, and the results confirmed that ERM treatment increased DEL-1 expression and suppressed bone loss by increasing the expression of osteoblast-associated factors. In conclusion, ERM restores bone metabolism homeostasis in inflammatory environments possibly via the induction of DEL-1.

## 1. Introduction

Periodontal disease is an infectious disease of the tissues surrounding the teeth and is characterized by alveolar bone resorption owing to inflammation caused by dysbiosis of the oral microflora, which ultimately leads to tooth loss [1]. Periodontopathogenic bacteria stimulate excessive migration of neutrophils into periodontal tissues and the secretion of inflammatory cytokines, thereby triggering inflammatory responses [2]. Furthermore, the local immune response to periodontal disease promotes bone loss by disrupting the homeostasis between bone formation and bone resorption [3]. Therefore, in periodontitis treatment, in addition to eliminating the infection, inflammation and bone metabolism mediated by osteoclasts and osteoblasts must be controlled. Accordingly, several new candidate substances, including flavonoids, rice peptides, and hinokitiol, have been reported to exert therapeutic effects on inflammation and bone resorption in periodontal disease [4,5,6,7]. However, no established approach exists for the treatment of periodontal disease that restores homeostasis in bone metabolism, including effects on bone formation and osteoblasts.

Macrolides are antibiotics that inhibit protein synthesis by targeting the bacterial ribosome and exhibit a broad spectrum of activity against bacteria [8]. Therefore, they are used to treat various infections such as pneumonia, skin infections, infectious enteritis, *Helicobacter pylori* infection, and periodontal disease [9,10,11,12,13]. In addition, macrolides have a wide range of immunomodulatory properties beyond suppression and stimulation [14,15]. Macrolide-induced immunomodulation represents a well-established therapeutic approach for chronic respiratory diseases, including chronic obstructive pulmonary disease, cystic fibrosis, and bronchiectasis [16]. Furthermore, several studies have reported that macrolides affect bone metabolism. Among macrolides, azithromycin (AZM) promotes wound healing by suppressing inflammation via immunomodulation in a mouse apical periodontitis model [17], and clarithromycin promotes bone formation in a rabbit cranial crown defect model when used in combination with β-tricalcium phosphate [18]. Furthermore, rapamycin, a macrolide used as an anticancer drug, has been suggested to promote osteoblast differentiation and new bone formation in a lipopolysaccharide (LPS)-induced inflammatory environment [19]. Thus, macrolides may contribute to the restoration of bone metabolic homeostasis by promoting bone formation. However, the mechanism by which macrolides affect bone metabolism, especially osteoblast activity, remains unclear.

We previously showed that developmental endothelial locus-1 (DEL-1) is induced by the immunomodulatory effects of macrolides and inhibits alveolar bone resorption by suppressing excessive neutrophil infiltration and osteoclast differentiation [20,21]. In addition, DEL-1 affects both osteoclasts and osteoblasts, thereby affecting bone metabolism. DEL-1 suppresses osteoclast differentiation by activating B-cell lymphoma 6 via interaction with Mac-1 integrin in osteoclasts and by downregulating nuclear factor of activated T-cells, cytoplasmic 1, which is a master transcription factor for osteoclast differentiation [22]. Furthermore, for osteoblastic progenitor cells, DEL-1 can activate the β3 integrin–focal adhesion kinase (FAK)–extracellular signal-regulated kinase 1 (ERK1)/2–runt-related transcription factor 2 (RUNX2) pathway and promote new bone formation in mice [23]. Based on these findings, we hypothesized that erythromycin (ERM) may not only inhibit osteoclast differentiation, but also promote osteoblast differentiation by inducing DEL-1, thereby restoring the homeostasis of bone metabolism that is lost in inflammatory conditions. The effects of ERM on osteoblast differentiation and bone formation activity were investigated both in vitro and in vivo.

The purpose of this study was to analyze ERM-mediated immunomodulatory effects on bone metabolism in periodontal inflammatory conditions. Periodontopathogenic bacteria have strong pathogenic properties that induce inflammatory reactions in periodontal tissues. A major periodontopathogenic bacterium is *Porphyromonas gingivalis*, a Gram-negative anaerobic bacterium [24]. *P. gingivalis* encodes various virulence factors such as LPS, fimbriae, hemagglutinin, and protease gingipains [25,26]. Among these, LPS exerts inhibitory effects on osteoblast differentiation and bone formation [27]. Furthermore, *P. gingivalis-*derived LPS promotes bone loss by suppressing osteoblast differentiation via TLR2-mediated and Notch1 signaling activation [28,29]. Hence, in this study, we investigated whether ERM could restore the inflammation-induced suppression of osteoblast differentiation and bone formation induced by *P. gingivalis-*derived LPS.

## 2. Results

### 2.1. ERM Ameliorated P. gingivalis LPS-Induced Decrease in Mineral Nodule Formation in MC3T3 Cells by Promoting Osteoblast Differentiation

**Figure 1:**
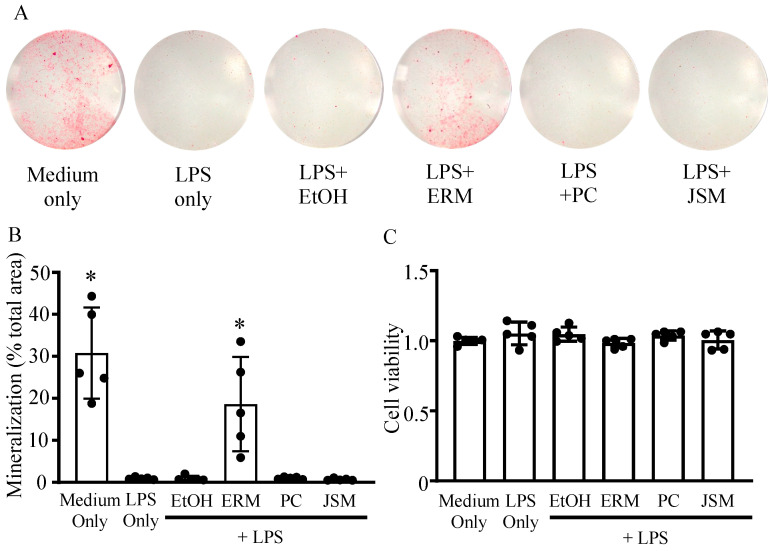
Erythromycin rescued osteoblast mineralization inhibited by *Porphyromonas gingivalis* LPS. MC3T3-E1 osteoblastic progenitors were incubated in growth medium with *P. gingivalis* LPS (100 ng/mL) and 20% ethanol (EtOH), erythromycin (ERM, 10 μg/mL), penicillin (PC, 5 unit/mL), or josamycin (JSM, 10 μg/mL). (**A**) Representative images (entire bottom of each well of a 96-well plate) of mineralized nodule formation detected by Alizarin Red S staining after 15 days. (**B**) The total mineralization area in each culture was quantified and expressed in % relative to the total area. (**C**) Cell viability was assessed using the MTT assay. * *p* < 0.05 compared to the EtOH + LPS group; values are shown as the mean ± SD (n = 5 per group).

First, we examined the effect of ERM on osteogenesis in vitro by culturing MC-3T3 cells in osteoblast differentiation medium containing *P. gingivalis* LPS. MC-3T3 cells have been cloned as osteoprogenitor murine cell lines and are used in several osteoblast differentiation and characterization experiments [30,31]. Furthermore, the properties of the mineral and matrix phases of MC3T3-E1 osteoblast cultures have been reported to be highly similar to those of mouse bones in terms of mineral structure and composition [32]. After MC3T3 cells were cultured with *P. gingivalis* LPS (100 ng/mL) for 15 days, Alizarin Red staining was performed to measure the amount of mineralized nodule formation. The amount of mineral nodule formation was substantially decreased in all *P. gingivalis* LPS-supplemented groups except the ERM (10 μg/mL) treatment group. In the LPS + ERM treatment group, nodule formation was restored to a level similar to that in the control group (Figure 1A,B). *P. gingivalis* LPS and the antibiotics did not affect the viability of MC-3T3 cells at the concentrations tested, as shown in the MTT assay (Figure 1C).

**Figure 2:**
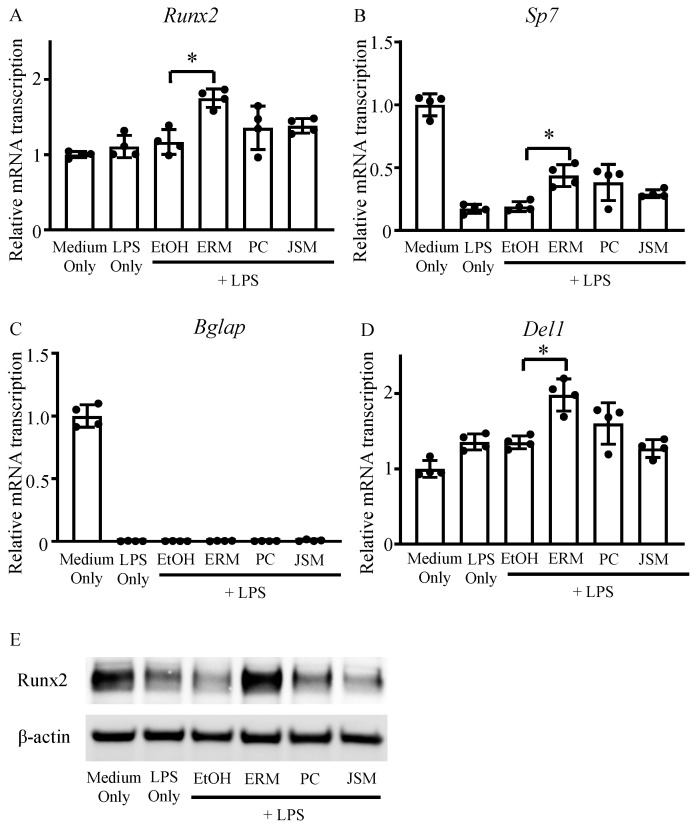
Erythromycin promoted the expression of DEL-1 and osteoblast differentiation-related factors. MC3T3-E1 osteoblastic progenitors were incubated in growth medium with *P. gingivalis* LPS (100 ng/mL) and EtOH, ERM (10 μg/mL), PC (5 unit/mL), or JSM (10 μg/mL). (**A**–**D**) The mRNA transcription levels of osteoblast differentiation-related factors and Del1 were quantified using real-time qPCR on day 12; * *p* < 0.05 compared to the EtOH + LPS group; values are shown as the mean ± SD (n = 4 per group). (**E**) Intracellular expression of Runx2 protein was determined using western blot analysis on day 12.

We measured the mRNA expression levels of osteoblast differentiation-related factors and *Del-1* on day 12 of culture. The mRNA expression levels of *Sp7* and *Bglap* were decreased in all LPS (100 ng/mL) groups compared to those in the control group (Figure 2B,C). In addition, *Runx2* and *Sp7* were markedly upregulated in the LPS + ERM (10 μg/mL) group compared to the EtOH + LPS group (Figure 2A,B). Notably, *Del1* was also strongly upregulated in the LPS + ERM group (Figure 2D). Furthermore, when the protein expression of RUNX2 in each group was analyzed by western blotting, it was found to be markedly decreased in the LPS-supplemented group, except in the LPS + ERM group (Figure 2E). These results suggest that *P. gingivalis* LPS severely suppressed the expression of factors essential for osteoblast differentiation and that ERM might attenuate this effect and restore osteoblast differentiation activity.

### 2.2. ERM Significantly Suppressed Periodontal Bone Loss Induced by P. gingivalis LPS

**Figure 3:**
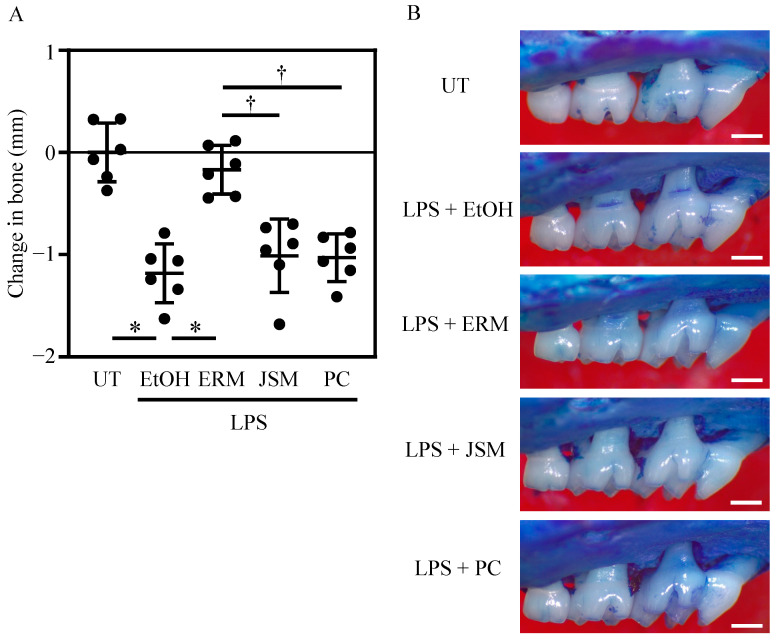
Peritoneal injection of antibiotics inhibits bone resorption in *Porphyromonas gingivalis* LPS-induced periodontitis. Periodontal bone loss was induced by the administration of *P. gingivalis* LPS to maxillary molars. The untreated (UT) group was set as a baseline control. The groups of mice were administered intraperitoneally 20% ethanol (EtOH; control), erythromycin (ERM), josamycin (JSM), or penicillin (PC). (**A**) The distance from the cement–enamel junction to the pinnacle of the alveolar bone was measured. Negative values (in mm) indicate bone loss relative to the UT control. (**B**) Representative images of the mouse maxillary bone from the indicated groups (scale bar of 0.5 mm). * *p* < 0.05 compared to the EtOH group; † *p* < 0.05 compared to the ERM group; values are shown as the mean ± SD (n = 5 per group).

Based on the aforementioned in vitro results, we analyzed the effect of ERM on *P. gingivalis* LPS-induced inflammatory alveolar bone resorption in vivo. To measure the immunomodulatory effects of antimicrobial agents, a mouse periodontitis model using *P. gingivalis* LPS was established. Several studies have reported the induction of alveolar bone resorption via local administration of *P. gingivalis* LPS to the gingiva of mice [33,34,35]. In the present study, considerable alveolar bone resorption was similarly induced via administration of *P. gingivalis* LPS (500 μg/kg/d). Analysis of the suppression of alveolar bone resorption in the antimicrobial treatment group showed that alveolar bone resorption was potently suppressed in the ERM (100 mg/kg/d) treatment group alone. The PC (10,000 unit/kg/d) and JSM (100 mg/kg/d) treatments did not suppress alveolar bone resorption (Figure 3A,B).

### 2.3. ERM Recovered the Expression of Osteoblast Differentiation-Related Factors Suppressed by P. gingivalis LPS

**Figure 4:**
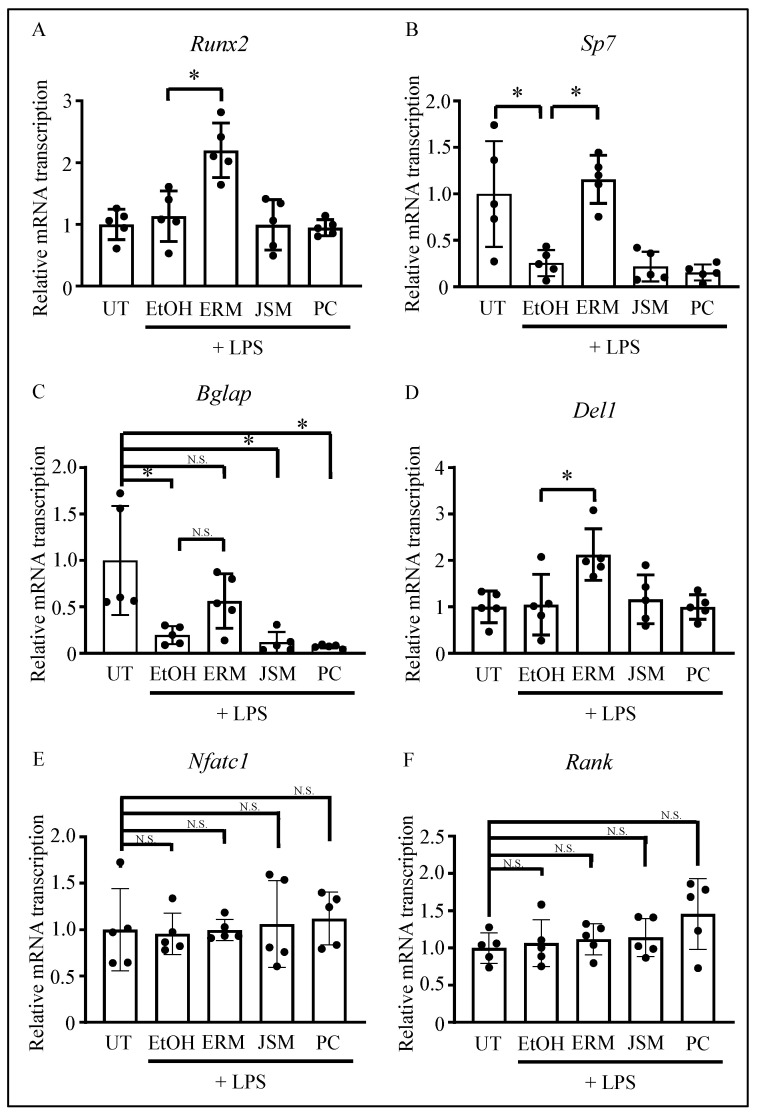
Effect of antibacterial drugs on the transcription of osteoblast-related factors in the gingiva, using the *Porphyromonas gingivalis* LPS-induced periodontitis model. (**A**–**F**) Real-time qPCR was performed to quantify the mRNA transcription levels of osteoblast-related factors, DEL-1, and osteoclast-related factors. * *p* < 0.05 compared to the indicated group; values are shown as the mean ± SD (n = 5 per group). N.S., not significant.

*Runx2*, *Sp7* (osterix), and *Bglap* are typically expressed as osteogenic markers in the early, middle, and late stages of osteoblast differentiation. Several studies have reported that LPS inhibits osteoblast differentiation via gene regulation [27,36,37]. Therefore, we measured gene expression levels in the palatal gingiva of mouse molars via qPCR. We found that *Sp7* and *Bglap* were substantially downregulated in all the *P. gingivalis* LPS-supplemented (500 μg/kg/d) groups except the ERM (100 mg/kg/d) treatment group. In contrast, the expression levels of *Runx2* and *Del1* were unaffected by the addition of *P. gingivalis* LPS. Furthermore, ERM treatment markedly upregulated *Runx2* and *Del1* compared to the levels measured in the EtOH group (Figure 4A–D). Therefore, ERM may facilitate the recovery from the downregulation of *Sp7* and *Bglap* induced by *P. gingivalis* LPS. In addition, osteoclast differentiation-related factors, which are crucial for bone resorption in periodontitis, were also analyzed. The results showed no significant differences in *Nfatc1* and *RANK* mRNA transcription between the groups (Figure 4E,F). These results of samples collected 2 weeks after LPS injection are consistent with the fact that in the periodontitis model with LPS administration, the strongest bone loss occurred within the first week of administration, followed by a further loss within one to two weeks, whereas there was little difference in bone levels between two and three weeks (Figure S1).

### 2.4. ERM Rescued DEL-1 Expression Reduced by P. gingivalis LPS

**Figure 5:**
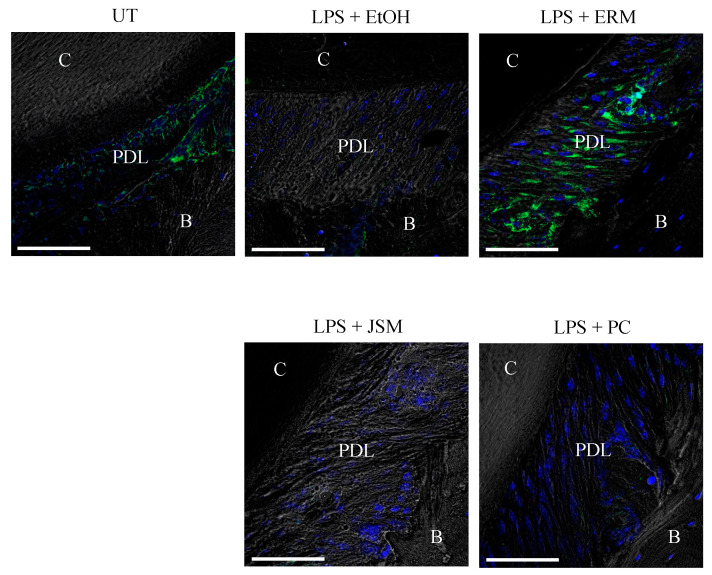
Erythromycin increases DEL-1 expression in the periodontal ligament. Frozen maxillae sections were stained for DEL-1 (green) and nuclei using DAPI (blue). Representative images obtained by optical microscopy are shown. C: cementum; PDL: periodontal ligament; B: alveolar bone. Scale bars, 50 µm.

DEL-1 protein expression in periodontal ligament tissues was examined via immunohistochemical analysis. The EtOH + LPS (500 μg/kg/d) group showed a decrease in DEL-1 protein expression, whereas the LPS + ERM (100 mg/kg/d) treatment group showed a rescue of DEL-1 protein expression in periodontal ligament tissues (Figure 5). This result was similar to that of the experiment in which ERM treatment was performed in a mouse model of tooth-ligated periodontitis [21].

### 2.5. ERM Enhanced Alkaline Phosphatase (ALP) Activity Attenuated by P. gingivalis LPS

**Figure 6:**
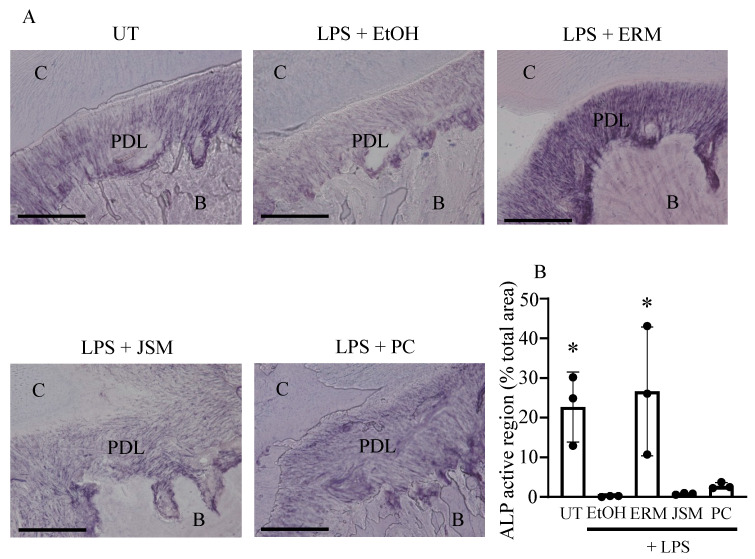
Erythromycin increases the alkaline phosphatase activity of cells in periodontal ligament tissue. Alkaline phosphatase staining was performed on frozen maxillary sections. (**A**) Representative images obtained by optical microscopy are shown. C: cementum; PDL: periodontal ligament; B: alveolar bone. Scale bars, 100 µm. (**B**) The total active region of alkaline phosphatase in each field was quantified and expressed as % relative to the total area. * *p* < 0.05 as compared to EtOH + LPS group, means ± SD (n = 3 per group).

ALP staining was performed to histologically evaluate the osteoblasts in periodontal tissues. In the EtOH + LPS (500 μg/kg/d) group, ALP activity in periodontal ligament tissues was attenuated when compared to that in the control group. In contrast, ALP activity was partially enhanced in periodontal ligament tissues in the LPS + ERM (100 mg/kg/d) treatment group (Figure 6).

## 3. Discussion

In this study, ERM upregulated *Del1* in vivo and in vitro and recovered osteoblast differentiation that was attenuated by *P. gingivalis* LPS-induced inflammation. Our results suggest that ERM has the ability to restore the homeostasis of bone metabolism in inflammatory environments by inducing DEL-1. Several studies have reported that macrolides are effective in the treatment of periodontal disease [13,38]. This has been attributed to the immunomodulatory effects of macrolides in addition to their antibacterial effects [39,40,41]. Macrolides have the characteristic structure of a macrocyclic lactone ring and are classified into several types based on differences in the structure [42]. Macrolides classified based on 14-membered rings (such as erythromycin and clarithromycin) and 15-membered rings (such as AZM) have immunomodulatory and anti-inflammatory properties, which are absent in 16-membered-ring macrolides (such as JSM) [15,43]. The results of the present study showed that ERM, a 14-membered-ring macrolide, has a strong inhibitory effect on inflammatory bone resorption induced by LPS, independent of bacterial infection. In addition, previous studies using a model of periodontitis with ligated teeth have shown similar results, which indicate that ERM has a strong inhibitory effect on bone resorption [20,21]. These findings suggest that macrolides suppress inflammation via immunomodulatory effects, at least in the entire tissue. In contrast, macrolides may elicit different immune responses in different cell types. AZM may alter the inflammatory response in human gingival fibroblasts by increasing the expression of interleukin 6 (IL-6) and IL-8 under LPS stimulation [44,45]. Thus, to elucidate the mechanism of macrolide-mediated immunomodulation, the effects of macrolides on different cell types must be analyzed. In the analysis of alveolar bone resorption due to the periodontitis, it is important to analyze both bone resorption by osteoclasts and bone formation by osteoblasts. In particular, only a few macrolide-derived effects on osteoblasts are known. Therefore, we focused on the effects of ERM on osteoblasts and collected samples 2 weeks after LPS and ERM injection, the optimal time to confirm osteoblast differentiation. Furthermore, at this point, the present experiment suggested that strong osteoclast differentiation did not occur, at least at the genetic level. Consequently, the present analysis focused more on osteoblasts. Considering the results of previous experiments, it is possible that erythromycin mainly acts on osteoclasts up to 1 week after the induction of periodontitis and then shifts its action to osteoblasts, contributing to the regulation of bone metabolism. Our study shows for the first time that ERM increases or restores the expression of osteoblast differentiation-related factors and ALP activity. ERM likely restored the homeostasis of bone metabolism by promoting osteoblast differentiation and bone formation under *P. gingivalis* LPS-induced inflammatory conditions.

The effect of LPS on osteoblasts has been analyzed in various aspects. LPS has been found to induce apoptosis when added to MC-3T3 cells at high concentrations [46]. In the present study, we focused on the effect of ERM on LPS-induced inhibition of osteoblast differentiation and therefore used a concentration of LPS that did not affect cell viability. The expression of *Runx2*, *Sp7*, and *Bglap*, which are essential for osteoblast differentiation, is repressed by *E. coli* LPS in vitro [47,48]. In the present study, the addition of *P. gingivalis* LPS to MC-3T3 cells markedly decreased the expression of *Runx2*, *Sp7*, and *Bglap*. These findings suggest that *P. gingivalis* and *E. coli* LPSs have an inhibitory effect on osteoblast differentiation and can contribute to the deterioration of tissues in periodontal pathology. *P. gingivalis* LPS treatment suppresses ALP activity and osteocalcin expression in human periodontal ligament cells and promotes the transition from bone formation to bone resorption in experiments using mouse osteoblasts and osteoclasts [49,50]. In addition, *P. gingivalis* LPS is known to have a characteristic toxic effect, inducing inflammatory responses via TLR2 in addition to inducing inflammation via TLR4 [51]. Furthermore, TLR2-mediated stimulation activates osteoclast differentiation by increasing receptor activator of nuclear factor kappa-Β ligand expression in osteoblasts [52], suggesting that *P. gingivalis* LPS stimulation activates bone resorption via its action on osteoblasts [53]. Subsequently, based on these molecular findings, the effects of ERM were analyzed in an animal model using *P. gingivalis* LPS, which also has unique toxic effects on bone metabolism. Several animal periodontal disease models using LPS have been established previously, including models using *P. gingivalis* LPS and *Aggregatibacter actinomycetemcomitans* LPS [54,55]. Currently, the tooth ligation model is commonly used as a mouse model for studying periodontal disease, and several studies have applied this model to analyze the molecular pathology of the disease and to investigate new treatment approaches [56,57]. However, in the tooth ligation model, alveolar bone resorption is induced by dysbiosis caused by the biofilm attached to the ligature threads [58,59]. Hence, the analysis of the inhibitory effect of antimicrobial agents on inflammatory alveolar bone resorption using the tooth ligation model cannot completely exclude the effect of antimicrobial properties. Therefore, in this study, we attempted to analyze the effects of ERM on bone formation based on its immunomodulatory effects using the *P. gingivalis* LPS-induced alveolar bone resorption model.

One mechanism by which ERM positively regulates osteoblast differentiation may involve DEL-1, which is a 52 kDa protein secreted by various tissue-resident cells, including endothelial cells, osteolineage cells, and certain macrophage subsets [60,61]. In addition, DEL-1 is a standard local regulator of tissue immunoplasticity and inflammatory disease, with its ability to promote macrophage efferocytosis to clear inflammation and activate regulatory T-cell function [62,63,64,65]. Bone healing and remodeling require the proper regulation of the inflammatory response, and while severe inflammation obviously inhibits bone regeneration, an appropriate level of inflammation-related factors is essential [66,67]. Furthermore, an anti-inflammatory treatment to restore the homeostasis of bone metabolism requires an immunomodulatory action, as studies indicate that the administration of anti-inflammatory drugs has a negative effect on bone healing [68,69]. 

Because DEL-1 has an important immunomodulatory effect on bone healing, DEL-1 may positively regulate bone metabolism in osteoclasts and osteoblasts as an effect on osteolineage cells. Particularly, DEL-1 inhibits osteoclast differentiation and bone resorption activity [22], whereas it promotes bone formation in osteoblasts; thus, DEL-1-deficient mice fail to regenerate bone unless recombinant DEL-1 is administered locally [23]. Hence, ERM might have strongly suppressed bone resorption by increasing DEL-1 expression in the periodontal tissue in this study. We previously reported that resolvin D1 and ERM suppress inflammatory bone resorption by inducing DEL-1 [70]. In addition, DEL-1 promotes osteogenic differentiation of osteoblastic cells in a β3 integrin-dependent manner. The ability of DEL-1 to promote in vitro osteogenesis, indicated by the induction of osteogenic genes such as the master transcription factor *Runx2* and by mineralized nodule formation, depends on its capacity to induce the phosphorylation of FAK and ERK1/2 [23]. Consequently, DEL-1 positively regulates osteoblast differentiation.

Certain limitations were noted in our current study. In this experiment, LPS injection and antimicrobial treatment were started at the same time. In other words, periodontitis was induced, and treatment was initiated simultaneously. However, because periodontal disease treatment in clinical practice is usually performed after the disease becomes apparent in the patient, not all of the results of this experiment can be considered translatable to actual clinical situations. Periodontal disease is a chronic inflammatory disease, and after the onset of the disease, the pathological process progresses through a series of quiescent and active phases [71]. In this experiment, LPS injection was used to induce acute inflammation, which can be regarded as a model that mimics the pathophysiology of periodontitis during the active phase of the disease. Therefore, the results of this study suggest that treatment with erythromycin during the active phase of periodontal disease could suppress the progression of the disease. In addition, there is a challenge in applying the results of this study to clinical practice. That is, the development of resistance to macrolide antimicrobial agents, including erythromycin, is a problem in the treatment of many infectious diseases [72,73,74,75]. In oral bacteria, the erythromycin resistance genes erm (B) and erm (F) were detected using PCR in oral *Prevotella* strains [76]. If macrolide antibiotics are used for a certain long period of time in anticipation of their bone metabolism-regulatory action, they may promote the growth of drug-resistant bacteria. Therefore, the development of macrolide molecules without antimicrobial activity is expected [77,78]. We expect that a more detailed clarification of the bone metabolism modulatory mechanism and regulatory molecules by erythromycin will lead to the development of macrolide-based bone metabolism agents that do not produce drug-resistant bacteria.

## 4. Materials and Methods

### 4.1. Reagents

The following antibacterial drugs were obtained: ERM (FUJIFILM Wako Pure Chemical Corporation, Tokyo, Japan), josamycin (JSM) (Sigma-Aldrich, St. Louis, MO, USA), and penicillin (PC) (Meiji Seika Pharma Co., Ltd., Tokyo, Japan). Each substance was dissolved in 80% phosphate-buffered saline (PBS) and 20% ethanol (EtOH).

### 4.2. Murine Model

C57BL/6Ncrl mice (age, 10 weeks; Charles River Laboratories Japan, Inc., Yokohama, Japan) were maintained in individually ventilated cages and provided sterile food and water ad libitum under specific pathogen-free conditions. All animal experiments were approved by the Institutional Animal Care and Use Committee of Niigata University (SA00181). The mice were housed at ambient temperature and humidity in ventilated caging systems on a 12 h/12 h light/dark cycle. Periodontitis was induced in mice via microinjection of *P. gingivalis*-derived LPS (500 μg/kg/d; InvivoGen, San Diego, CA, USA) into the palatal gingiva once a day for 2 weeks. Antibacterial drugs (ERM, 100 mg/kg body weight; PC, 10,000 unit/kg body weight; JSM, 100 mg/kg body weight) or 20% EtOH were administered intraperitoneally once a day for 14 days in the intervention experiments. The mice were euthanized 14 days after the start of *P. gingivalis* LPS administration. The gingiva was dissected and processed for performing real-time quantitative polymerase chain reaction (qPCR) determination of osteoblast-related factors and *Del1* mRNA expression levels. Periodontal bone loss was morphometrically assessed in defleshed maxillae using a stereoscopic microscope (Leica Microsystems, Wetzlar, Germany) (35×). The distance from the cement–enamel junction to the alveolar bone crest (CEJ-ABC) was measured in 13 sites at predetermined points from the first molar to the third molar. Bone loss was calculated by subtracting the sum of the values at the 13 sites from CEJ-ABC in the untreated regions. Negative values (mm) indicated bone loss relative to the baseline (untreated control).

### 4.3. Histological Analysis

For the standard histological and subsequent quantitative histomorphometric analyses, the maxillae were fixed in 4% paraformaldehyde (PFA)-containing PBS (Wako Pure Chemical Industries, Osaka, Japan) for 24 h, followed by decalcification in Decalcifying Solution B (Wako Pure Chemical Industries) for 1 week at 4 °C. The specimens were then embedded in optimal cutting temperature compound (Sakura Finetek, Torrance, CA, USA) and frozen in liquid nitrogen, and coronal sections were cut using a cryostat (Leica Biosystems, Wetzlar, Germany).

### 4.4. Osteoblastic Progenitors

The murine osteoblastic progenitor cell line MC3T3-E1 was obtained from the RIKEN Bioresource Center (RCB1126). MC3T3-E1 cells were maintained in minimum essential Eagle medium, alpha modification (α-MEM; FUJIFILM Wako Pure Chemical CorporationOsaka, Japan), supplemented with 10% fetal bovine serum (FBS; SERANA Brandenburg, Germany), a penicillin–streptomycin solution (×100) (FUJIFILM Wako Pure Chemical Corporation) at 37 °C, and 5% CO_2_.

### 4.5. Osteogenic Differentiation Assay

For osteogenic differentiation, osteoblast progenitor cells were cultured in α-MEM supplemented with 10% FBS and an osteoblast-inducer reagent (Takara Bio Inc., Kusatsu, Japan) for up to 15 days. Antibiotics and *P. gingivalis*-derived LPS were added to the aforementioned osteoblast differentiation medium and replaced every 3 days. Mineralized bone nodules were detected by staining with Alizarin Red S (FUJIFILM Wako Pure Chemical Corporation). Briefly, the cultures were washed twice with PBS, fixed with 4% PFA in PBS for 10 min, and washed again with distilled water. Staining was performed by immersing the cells in a 2% Alizarin Red S solution for 30 min. After washing with distilled water to remove the unbound dye, the entire well containing the calcified nodule was photographed. The calcified nodules were quantified as a percentage of area covered relative to the total area using ImageJ software (National Institutes of Health, Bethesda, MD, USA). Cell viability was assessed by MTT assay, in which MTT (400 µg/mL) was directly added to the cultures, followed by incubation at 37 °C and 5% CO_2_ in humidified air. Subsequently, the supernatant was aspirated and 200 µL of lysis solution (90% isopropanol, 0.5% SDS, 0.04 N HCl, DW) was added to dissolve the formazan dye. Optical density (OD) was measured at 570 nm using a microplate reader. The mean OD of the control group was set as 1, and the experimental groups were compared to the control.

### 4.6. Quantitative Real-Time PCR

Total RNA was extracted from MC3T3-E1 cells cultured in osteoblast differentiation medium for 12 days using the same method as described above and mouse maxillary palatal gingiva using the TRI reagent (Molecular Research Center, Inc., Cincinnati, OH, USA); RNA was quantified via spectrophotometry at 260 and 280 nm. RNA was reverse-transcribed using SuperScript VILO Ⅳ Master Mix (Thermo Fisher Scientific, Waltham, MA, USA). qPCR was performed using the cDNA in a StepOnePlus real-time PCR system (Thermo Fisher Scientific) according to the manufacturer’s protocol. The data were analyzed using the comparative CT (ΔΔCt) method. TaqMan probes, sense primers, and antisense primers for the expression of a housekeeping gene (glyceraldehyde 3-phosphate dehydrogenase, assay ID: 4331182 Mm99999915_g1), along with *Runx2* (assay ID: 4331182 Mm00501584_m1), *Sp7* (osterix, assay ID: 4331182 Mm00504574_m1), bone γ-carboxyglutamic acid (*Bglap*, assay ID: 4331182 Mm00649782_gH), epidermal growth factor-like repeats and discoidin domains 3 (*Edil3*; *Del1*, encoded DEL-1, assay ID: 4331182 Mm01291247_m1), nuclear factor of activated T cells 1 (*Nfatc1*, assay ID: 4331182 Mm01265944_m1), and tumor necrosis factor receptor superfamily member 11A (Tnfrsf11a; *Rank*, encoded RANK, assay ID: 4331182 Mm00437132_m1) were purchased from Thermo Fisher Scientific.

### 4.7. Western Blot Analysis

Proteins were extracted from lysates of MC3T3-E1 cells cultured in osteoblast differentiation medium for 12 days via the same method described above using M-PER Mammalian Protein Extraction Reagent (Thermo Fisher Scientific) with Halt Protease and Phosphatase Inhibitor Single-Use Cocktail (100×) (Thermo Fisher Scientific). The protein solution was centrifuged, and the supernatant was mixed with 4× Bolt LDS Sample Buffer (Thermo Fisher Scientific), separated by sodium dodecyl sulfate-polyacrylamide gel electrophoresis) on a Bolt 4–12% Bis-Tris Plus gel (Thermo Fisher Scientific), and transferred to a polyvinylidene difluoride Membrane Filter Paper Sandwich (Thermo Fisher Scientific). The membranes were probed using an anti-RUNX2 antibody (Abcam, Cambridge, UK) and incubated with a horseradish peroxidase (HRP)-conjugated secondary antibody (Cell Signaling Technology, Danvers, MA, USA) or an anti-β-actin HRP-conjugated antibody (Cell Signaling Technology), followed by incubation with StartingBlock (TBS) Blocking Buffer (Thermo Fisher Scientific). Subsequently, the membranes were incubated with ECL Select reagent (GE Healthcare, Little Chalfont, UK) and analyzed using an Image Quant LAS 4000 system (GE Healthcare Bio-Sciences AB, Uppsala, Sweden).

### 4.8. Statistical Analysis

The data were evaluated using analysis of variance (ANOVA) and one-way ANOVA with Tukey’s multiple comparisons test using GraphPad Software version 7.03 (GraphPad Software Inc., La Jolla, CA, USA). Statistical significance was set at *p* < 0.05.

## Data Availability

The original contributions presented in the study are included in the article, and further inquiries can be directed to the corresponding author.

## Supplementary Materials

The following supporting information can be downloaded at: https://www.mdpi.com/article/10.3390/ph16020303/s1, Figure S1: Bone resorption in *Porphyromonas gingivalis* LPS-induced periodontitis.
