# Supplementary material for: Erythromycin Restores Osteoblast Differentiation and Osteogenesis Suppressed by Porphyromonas gingivalis Lipopolysaccharide
Source: Pharmaceuticals (Basel). 2023 Feb 15;16(2):303. doi: 10.3390/ph16020303 (PMC9959121; doi:10.3390/ph16020303)
Supplement: Supplementary file 1 [file pharmaceuticals-16-00303-s001.zip › pharmaceuticals-2190938 - Figure S1.pdf]

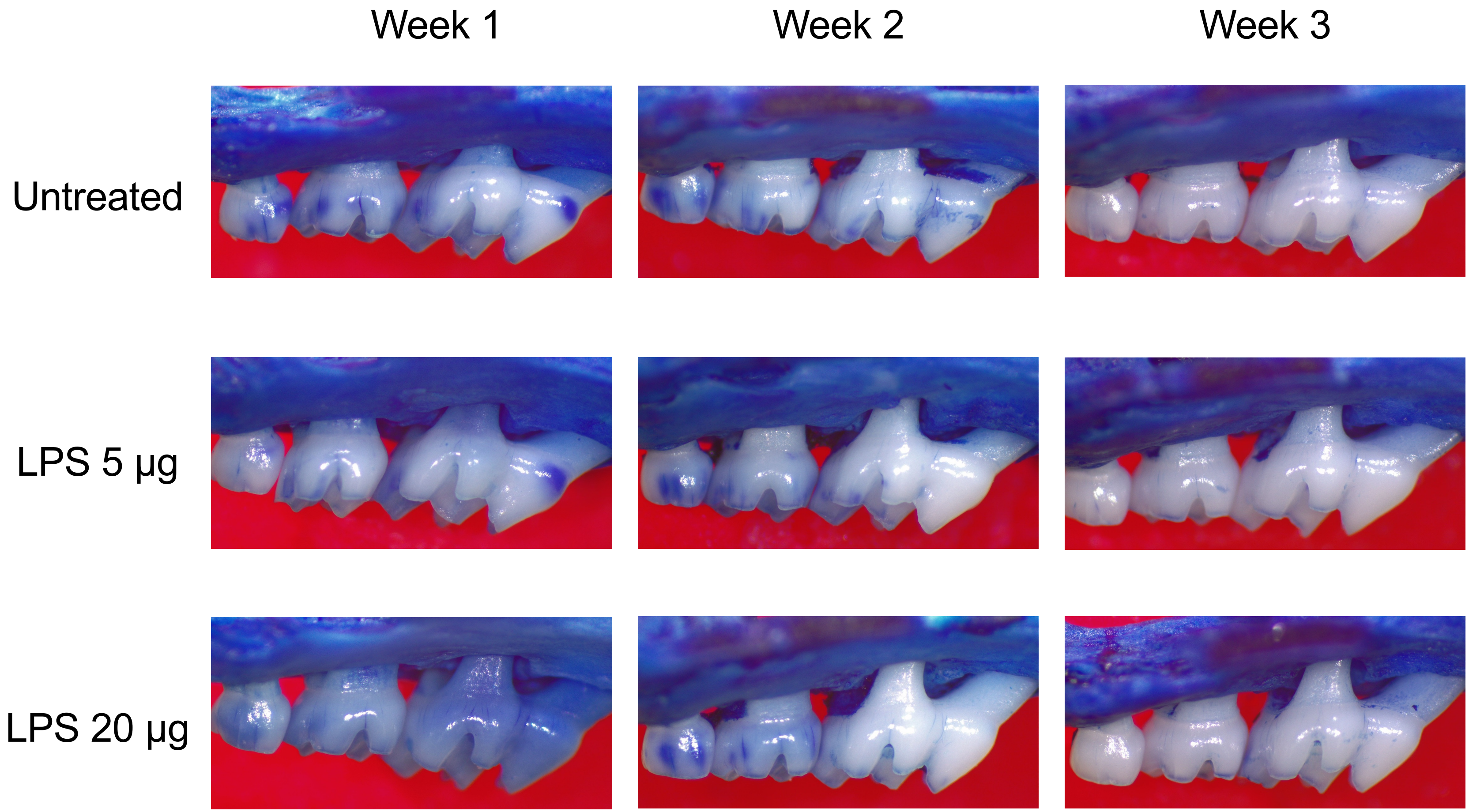

Figure S1. Bone resorption in *Porphyromonas gingivalis* LPS-induced periodontitis. Periodontal bone loss was induced by administration of *Porphyromonas gingivalis* LPS to maxillary molars. Representative images of mouse maxilla for each dose and time period.
