# Supplementary material for: Erythromycin Restores Osteoblast Differentiation and Osteogenesis Suppressed by Porphyromonas gingivalis Lipopolysaccharide
Source: Pharmaceuticals (Basel). 2023 Feb 15;16(2):303. doi: 10.3390/ph16020303 (PMC9959121; doi:10.3390/ph16020303)
Supplement: Supplementary file 1 [file pharmaceuticals-16-00303-s001.zip › Supplemental Materials and Methods.pdf]

Supplemental Materials & Methods

S1. Murine Model

Periodontitis was induced in mice via microinjection of *P. gingivalis*-derived LPS (250 or 1000 μg/kg/d; InvivoGen, San Diego, CA, USA) into the palatal gingiva once a day for 1, 2, or 3 wks. Antibacterial drugs (ERM, 100 mg/kg body weight; PC, 10,000 unit/kg body weight; JSM, 100 mg/kg body weight) or 20% EtOH were administered intraperitoneally once a day for 1, 2, or 3 wks in the intervention experiments. The mice were euthanized 1, 2, or 3 wks after the start of *P. gingivalis* LPS administration.
